# Case-Based Diagnostic Performance, Challenges and Tool Development in Identifying Orofacial Conditions Among Pediatric Dentists: A Mixed-Methods Study

**DOI:** 10.3390/diagnostics16193218

**Published:** 2026-10-04

**Authors:** Amani Sait, Nada O. Bamashmous, Khlood Baghlaf, Khalid Aljohani, Rahaf Sahhaf

**Affiliations:** 1 Pediatric Dentistry Department, Ministry of Health, Madina 42351, Saudi Arabia; asait0036@stu.kau.edu.sa; 2 Pediatric Dentistry Department, Faculty of Dentistry, King Abdulaziz University, Jeddah 21589, Saudi Arabia; nobamashmous@kau.edu.sa (N.O.B.); rsahhaf@kau.edu.sa (R.S.); 3 Department of Oral Diagnostic Sciences, Faculty of Dentistry, King Abdulaziz University, Jeddah 21589, Saudi Arabia; koalgehani@kau.edu.sa

**Keywords:** tool, diagnostic, performance, orofacial, children

## Abstract

**Background/Objectives:** This study assessed diagnostic performance, explored diagnostic barriers, and developed an assessment tool for case-based diagnostic performance and associated challenges specific to pediatric OFCs. **Methods:** An exploratory sequential mixed-methods study was conducted. The qualitative phase included semi-structured interviews with Saudi pediatric dentists, selected using criterion (purposive) sampling until thematic saturation was reached. Findings informed the development and validation of a structured questionnaire, targeting pediatric dentists from various regions of Saudi Arabia. The questionnaire assessed case-based diagnostic performance, appropriate management, and diagnostic barriers. Descriptive statistics were performed using SPSS. Findings from both phases were triangulated. **Results:** Thirteen interviews were conducted with pediatric dentists (specialists and consultants). Themes and subthemes, including key barriers, were identified. Among 180 pediatric dentists who answered the questionnaire, diagnostic performance was high, with mean diagnosis and management scores of 9.03 ± 1.16 and 8.84 ± 1.32, respectively. Triangulation demonstrated convergence regarding the importance of clinical exposure and interdisciplinary collaboration in improving diagnostic competence. **Conclusions:** The study developed a valid assessment tool specific to pediatric OFCs in Saudi Arabia, which may have limited generalizability to other countries. Pediatric dentists demonstrated high diagnostic performance and management competency, while key diagnostic barriers highlighted the need for enhanced hands-on training and interdisciplinary collaboration to further support accurate diagnosis and effective management of pediatric OFCs.

## 1. Introduction

Orofacial conditions (OFCs) are frequently encountered in children and adolescents and include a diverse range of conditions, such as neoplasms, hyperplastic lesions, reactive lesions, salivary gland abnormalities, bone lesions, and oral mucosal lesions [1]. These lesions can significantly impact physical functioning, psychosocial well-being, and overall quality of life [2,3].

Several retrospective studies conducted across different geographic locations have reported the prevalence of OFCs. In Poland, Owczarek-Drabińska et al. analyzed 2747 charts from 143 pediatric patients, ranging from 0 to 17 years of age, finding that aphthous ulcers were the most common lesion (18.9%), followed by mucoceles (10.5%), geographic tongue (5.6%), papilloma (4.9%), pyogenic granulomas (4.2%), and traumatic erosions and ulcers (4.2%) [4]. A study by Aly et al. involved a sample of 1108 specimens from Egyptian pediatric patients aged under 18 years and identified the most prevalent lesions: inflammatory radicular cysts (18.8%), followed by pyogenic granulomas (14.4%), peripheral giant cell granulomas (8.9%), irritational fibromas (8.1%), mucoceles (5.1%), periapical granulomas (4.0%), central giant cell lesions (3.7%), and dentigerous cysts (3.4%) [5]. Additionally, two studies conducted in Brazil revealed similar findings. Costa et al. analyzed 1480 biopsies from pediatric patients under 14 years of age, while Rezende et al. reviewed 93,950 reports from patients aged 0 to 3 years [6,7]. Both studies found mucoceles to be the most common lesions, occurring in 13.6% and 12% of cases, respectively. Similarly, a study conducted by Binmadi and Aldehlawi in Saudi Arabia found that mucoceles (12%) were more prevalent, with a notable predominance in females. The study included 183 pediatric cases of oral pathologies in patients aged 0 to 18 years. Periapical granulomas (7.7%) ranked second, followed by dentigerous cysts (6.6%), radicular cysts (6.6%), and pyogenic granulomas (4.9%) [8].

Pediatric dentists face many challenges in diagnosing OFCs. Recent literature highlights that oral ulcers are highly nonspecific symptoms with a wide range of potential causes, including infectious diseases, nutritional deficiencies, autoimmune disorders, medication side effects, and systemic diseases. This diversity of etiological factors complicates the diagnostic process [9]. A study conducted in Brazil evaluated the clinicopathological features of 315 oral lesions in 307 patients under the age of 18, revealing that only 61.5% of clinical hypotheses aligned with histopathological findings [10]. This indicates that nearly 40% of lesions were misdiagnosed or underdiagnosed based solely on clinical assessment, often due to a lack of adequate clinical and radiographic information. Additionally, challenges such as children’s developing anatomy, patient cooperation issues, and limitations in performing invasive diagnostics or obtaining imaging further hinder accurate diagnosis [11]. Other challenges related to dentists include limited access to oral medicine consultants within the dental institute [12].

To date, there are no available tools to investigate the diagnostic performance and challenges faced by dentists in diagnosing OFCs. Therefore, this study aimed to assess the diagnostic performance of pediatric dentists in identifying OFCs, explore the common barriers and challenges encountered during diagnosis, and develop an assessment tool to evaluate diagnostic performance and associated diagnostic challenges.

## 2. Materials and Methods

### 2.1. Ethical Considerations

Ethical approval for this research was obtained from the Research Ethics Committee at the Faculty of Dentistry at King Abdulaziz University (136-11-24; approved on 16 January 2025). Written consent was obtained from all pediatric dentists participating in each phase of the study.

### 2.2. Study Design

**Figure 1:**
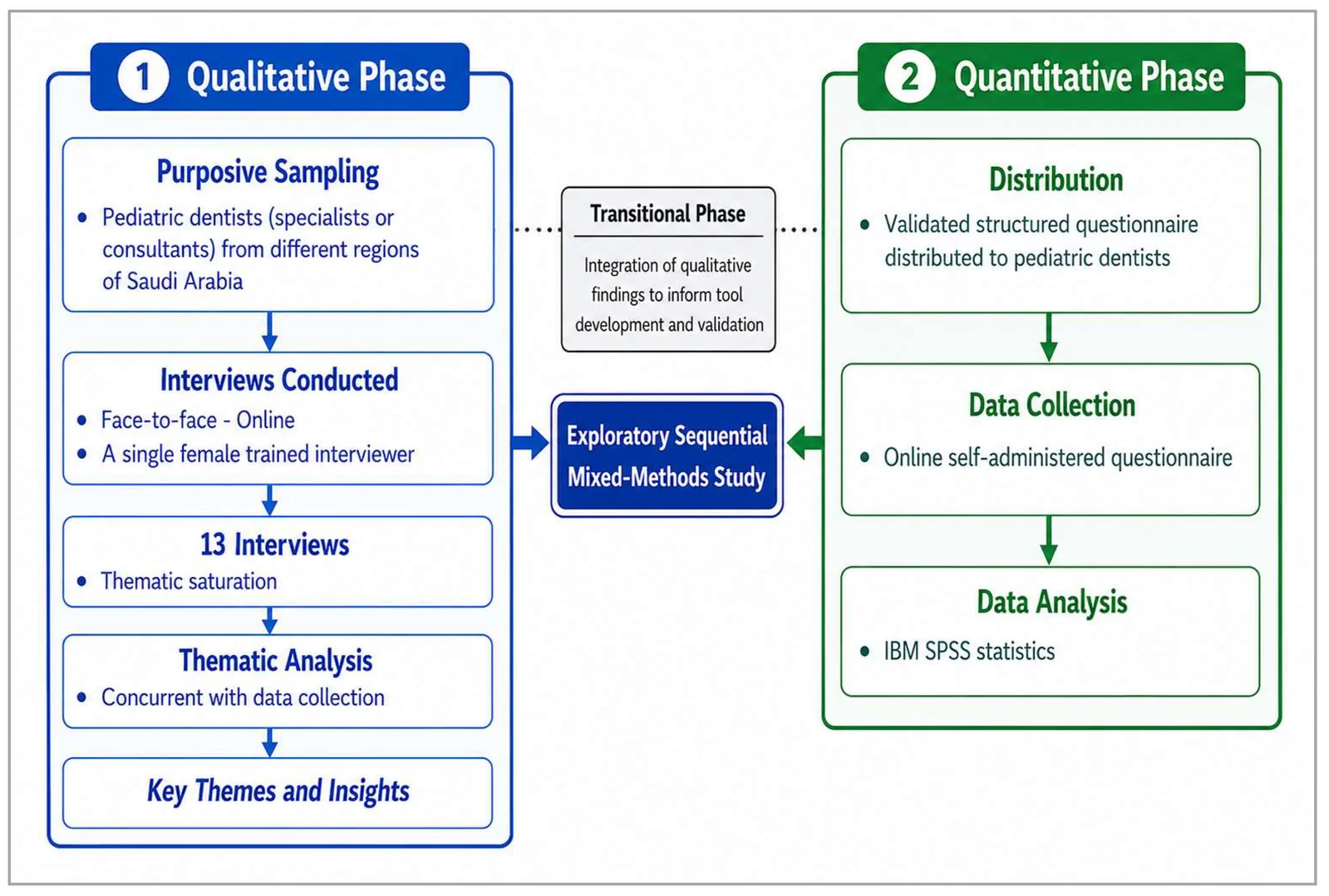
A mixed-methods study design process flowchart.

This study used an exploratory mixed-methods design that had two sequential phases. A mixed-methods study design flowchart is shown in Figure 1.

#### 2.2.1. Qualitative Phase (I)

##### Phase Design

A phenomenological qualitative phase was conducted to understand pediatric dentists’ knowledge and experiences regarding OFC cases and the challenges faced during their diagnosis and management.

##### Eligibility Criteria

Licensed pediatric dentists (either specialists or consultants) of both genders from various centers, including educational institutions, private clinics, and hospitals across multiple cities in Saudi Arabia, who had encountered OFCs within the past year and who consented to participate were included. Participants were required to be proficient in English. Rare cases and OFCs associated with syndromes were excluded.

##### Participants and Data Collection Procedure

A criterion (purposive) sample of pediatric dentists who met the inclusion criteria was invited to participate in semi-structured, audio-recorded interviews via WhatsApp. Participants were contacted in advance to provide a brief overview of the study, and a reminder message was sent 10 min before the interview. The semi-structured interviews were conducted either face-to-face or online via Google Meet (Google LLC, Mountain View, CA, USA), a web-based video-conferencing platform. Available at: https://meet.google.com/ (accessed on 2 January 2026), depending on participants’ preferences, with reminder texts sent 10 to 15 min prior to the interviews. The interviews were conducted in English using a standardized interview guide (Appendix A) consisting of 19 open-ended questions. The interviews lasted between 30 and 55 min. To ensure consistency, a single female resident in pediatric dentistry (Author 1) conducted all interviews. The resident was trained in qualitative research and was observed by a senior research expert in qualitative studies. Participants were asked the same questions in the same sequence, though the interviewer adapted probing questions based on participants’ key responses. The first interview was conducted as a pilot interview with a pediatric dentistry specialist. Minor modifications were made to the interview guide based on the pilot findings. The pilot interview was included in the analysis. Notes were recorded following each interview, and verbatim transcription was completed using transcription services. Data collection occurred from October 2025 to January 2026.

##### Sample Size

The sample size was determined by data saturation. Previous studies [13,14] suggest that 12–14 interviews are sufficient to achieve data saturation.

##### Data Analysis

Data analysis was conducted concurrently after each interview to monitor emerging themes until thematic saturation was achieved. Qualitative data from the verbatim transcripts were analyzed using NVivo software (version 15; Denver, CO, USA: Lumivero, 2024) to support coding, data organization, and theme development. Data analysis was performed by two coders (A.S. and K.B.), and an audit trail was used. Member checking was conducted. The COREQ (Consolidated Criteria for Reporting Qualitative Research) checklist was followed for reporting this study [15].

#### 2.2.2. Questionnaire Development and Validation

A structured, self-administered questionnaire was developed based on the qualitative phase, with the identified general and challenge-related themes directly informing its content. General themes guided items on the frequency of encountering OFCs, training received, curriculum, and nature of exposure, while opinions, recommendations, and challenges were incorporated based on the corresponding qualitative findings and themes. The common OFCs identified in phase I were compared with the existing literature, verified, and used to develop case scenarios. The qualitative findings also informed the relevant response options, while demographic items were adapted from previously published surveys. The questionnaire content was divided into three sections (Appendix B). The first section included sixteen items related to general demographic and professional characteristics. The second section included 10 case scenarios of common OFCs identified from the interviews and verified based on the existing literature [4,8,9], with 20 case-based items. For each case, participants were asked questions related to diagnosis and management. The third section included five items exploring the challenges faced by pediatric dentists when managing such cases, as well as their opinions and decision-making processes in diagnosis and management.

The questionnaire underwent content validation by five experts from King Abdulaziz University to assess its relevance, feasibility, practicality, validity, and clarity of interpretation: one associate professor in oral medicine and four associate professors in pediatric dentistry. Each item was evaluated by the five experts using a four-point scale, with a rating of 4 indicating high content relevance. The Content Validity Index (CVI) for each item was calculated as the proportion of experts assigning a rating of 4 (i.e., the number of experts who rated the item as 4 divided by the total number of experts). The overall scale-level CVI was calculated as the mean of the item-level CVI values, yielding an overall CVI of 0.82. Items with high agreement scores (rated 4) were retained, while items with lower agreement were revised or removed based on the experts’ feedback.

The questionnaire was then digitized using Google Forms and pilot-tested for face validity among five participants in pediatric dentistry before distribution, including two residents, two specialists, and one faculty member. The participants assessed the questionnaire for clarity and ease of completion. Minor modifications were made based on their feedback. The pilot participants were excluded from the main study.

#### 2.2.3. Quantitative Phase (II)

##### Phase Design

This phase involved the distribution of the validated questionnaire and subsequent collection and analysis of the quantitative data.

##### Eligibility Criteria

Pediatric dentists, including consultants, specialists, and residents enrolled in pediatric dentistry programs, from different regions across Saudi Arabia who were actively practicing within the country, were included.

##### Participants and Procedure

A non-probability maximum variation convenience sampling technique was employed in this phase to capture diverse perspectives and ensure representation of participants with varying backgrounds and characteristics. The final version of the questionnaire was distributed to participants through the WhatsApp and LinkedIn platforms. To enhance the response rate, multiple follow-up reminders were sent periodically to potential participants throughout the data collection phase. Data collection was conducted from the beginning of March 2026 to the end of May 2026. The questionnaire required approximately five to seven minutes to complete.

##### Sample Size

No statistical sample-size calculation was performed. The target population was estimated based on the reported 257 Saudi pediatric dentists registered with the Saudi Commission for Health Specialties (SCFHS) across Saudi Arabia in 2019 [16]. All accessible pediatric dentists who met the eligibility criteria were invited to participate. A 70% response rate was anticipated based on a previous study [17] and was used for recruitment planning only.

##### Data Analysis

Data were analyzed using IBM SPSS Statistics version 27 (IBM Corp., Armonk, NY, USA). Descriptive statistics were used to summarize participant characteristics and questionnaire responses. Continuous variables were presented as means and standard deviations (SD), while categorical variables were presented as frequencies and percentages.

Diagnostic and management scores were calculated separately for each participant as the number of correct responses across the 10 clinical cases, with each score ranging from 0 to 10. For the multiple-response questions, participants could select more than one option; therefore, responses were analyzed separately for each option and presented as frequencies and percentages based on the number of participants selecting each option.

## 3. Results

### 3.1. Results of Qualitative Phase (I)

#### 3.1.1. Sample Characteristics

A total of 13 interviews were conducted with pediatric dentists (Table 1). Of the 15 invited participants, two declined to participate, indicating that the interview approach was unsuitable for them. Seven interviews were conducted with consultants and six with specialists. Ten participants (76.9%) graduated from Saudi Arabia. More than two-thirds (nine participants; 69.2%) held board certification. The majority (11 participants; 84.6%) were female. Two-thirds (nine participants; 69.2%) worked in Jeddah, while four participants (30.8%) worked in other cities, including Jazan, Riyadh, Al Khobar, and Tabuk. Almost half (seven participants; 53.8%) had five to ten years of experience.

#### 3.1.2. Resulting General Themes

Following thematic analysis of the transcribed semi-structured interviews, nine themes and seven subthemes emerged regarding barriers faced by pediatric dentists in diagnosing OFCs.

**Figure 2:**
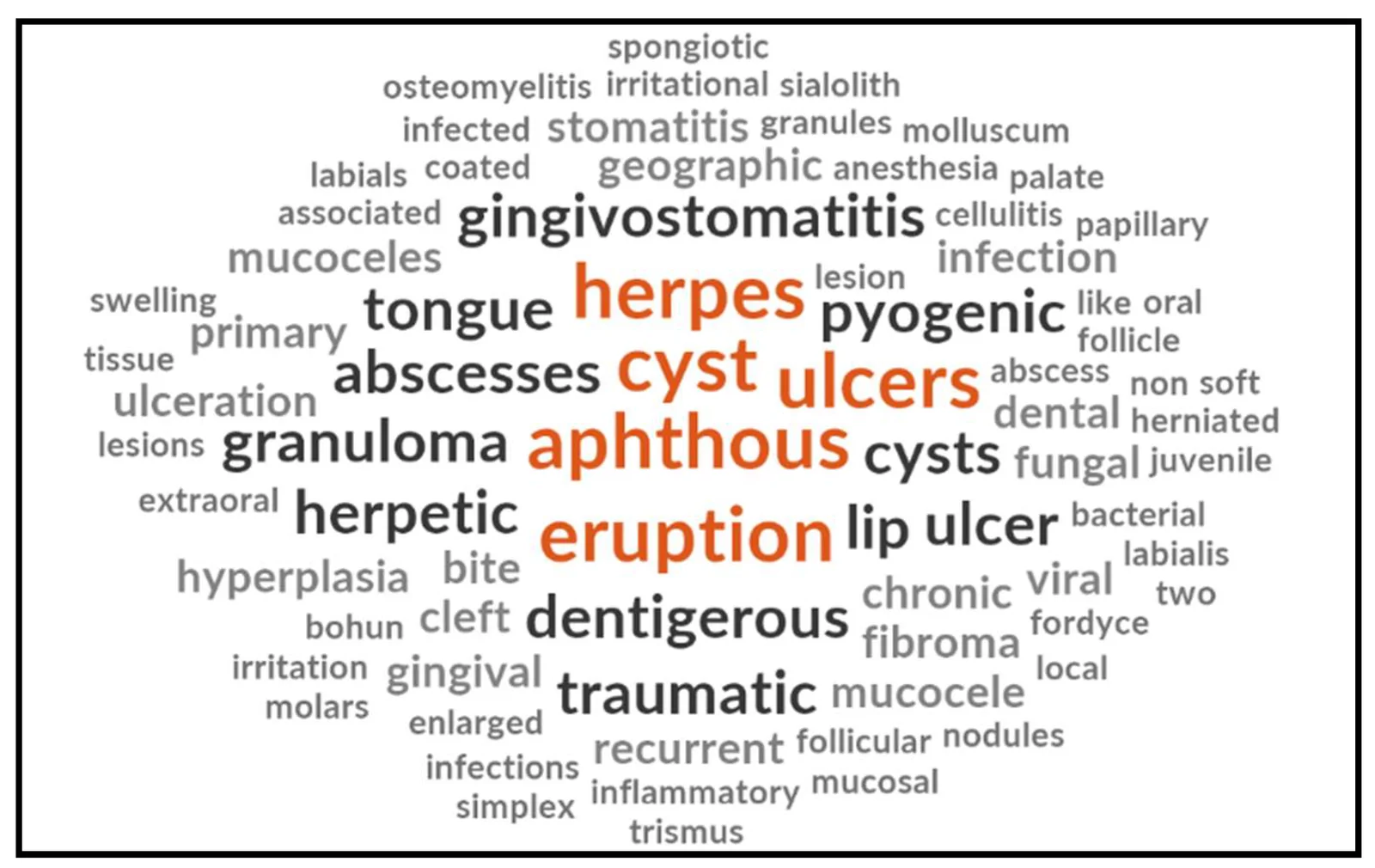
Word cloud of the most common OFCs reported by pediatric dentists.

Words from the most frequently reported OFCs in children by pediatric dentists over the last 12 months are presented in Figure 2.

##### Theme 1: Diagnostic Competence

For the case scenario representing severe primary herpetic gingivostomatitis, including a comprehensive history and detailed description of the clinical presentation, some participants provided the correct diagnosis, while others provided incorrect diagnoses, included the correct condition in their differential diagnoses, or were unable to make a diagnosis at all. The selected quote is listed below:

“*This needs a differential. Some sort of ulcerative disease could be considered. It could be leukoplakia, pemphigus vulgaris, or any other ulcerative disorder. I would do further investigations, of course. I would ask about Behçet’s, allergies, it could also be from the appliance.*”

[Participant 2 N C]

Table 2 shows that specialists and consultants were represented equally. Overall, the findings indicate that diagnostic performance was not 100%, highlighting variability in case evaluation among participants.

##### Theme 2: Diagnostic Methods

The most frequently reported diagnostic approaches included patient history, clinical examination, and imaging (e.g., cone-beam computed tomography [CBCT]), depending on the presenting condition. The following quotation illustrates how participants approached the diagnostic process and reached a final diagnosis:

“*There was no chief complaint. It was an incidental radiographic finding. We took a panoramic radiograph, conducted a clinical examination, requested a CBCT, consulted with the surgery team, and we will take an aspiration biopsy to reach the final diagnosis. We put a differential diagnosis of a dentigerous cyst and OKC.*”

[Participant 7 S C]

##### Theme 3: Frequently Encountered Orofacial Conditions

Participants reported variation in the frequency with which they encountered pediatric OFCs in clinical practice. While some participants reported seeing these conditions relatively frequently, others described these cases as rare and encountered them only sporadically. This is reflected in the following participant quotations:

“*Very frequently, actually. Once in every other week maybe, once one in every two weeks I receive a case that relates to oral lesions.*”

[Participants 10 A S]

“*Very rare honestly, how many do we see? it’s not daily or weekly. It’s monthly. You know, every month you can say we can see one case, for example. Sometimes more than one month.*”

[Participants 5 O C]

##### Theme 4: System of Referral

Most pediatric dentists reported that they initiate internal referrals through the institutional electronic-based system and adhere strictly to established hospital protocols. However, many participants emphasized that, beyond the formal referral pathway, they prefer to directly contact the receiving physician or department by phone. This step was described as a strategy to prevent delays, minimize the risk of patients being “lost” within the system, and ensure timely coordination of care.

“*I work in a place where we have many other medical specialties, and this would involve an internal referral system through the hospital. However, in private practice, I refer patients to my colleague in dentistry. This would be either a direct referral or a written referral. So, it honestly depends on the case.*”

[Participant 10 A S]

##### Theme 5: Nature of Curriculum Training

Most participants reported that their residency training included education in oral pathology or oral medicine. While some participants described receiving primarily didactic courses, others emphasized that their training was reinforced by extensive clinical exposure to patients with orofacial lesions. Participants described their experiences as follows:

“*My program training was a tertiary care center. It was heavy loads of patients. so, we tend to see lots of lesions. Because you know, the incidence of oral pathology, per se, is not much. The incidence of general oral diseases is not much. So, in our training, we used to have heavy loads of patients, so we tend to see a little bit more lesions than others.*”

[Participant 2 N C]

#### 3.1.3. Resulting Subthemes of Challenges Faced by Pediatric Dentists

A. Dentist-Related Themes

1. Insufficient Training or Experience

One perspective expressed by a participant emphasized that insufficient training in residency programs, as well as limited exposure at the undergraduate level, contributes to gaps in clinical experience and presents challenges in diagnosing and managing orofacial lesions, often leading to referrals to the oral medicine department.

“*Maybe all of what we studied as didactic in the book, including the cases in the photos, is relevant, but I definitely mean the challenge that we don’t see it in the clinic, so it doesn’t get reinforced.*”

[Participant 9 I C]

B. Lesion-Related Themes

2. Complexity and Atypical Presentation of Orofacial Lesions

Many participants highlighted the challenges posed by the variability of oral lesions, noting that even the same type of lesion can differ significantly, sometimes making it difficult to identify. Some quotes related to this theme are listed below:

“*The main challenges any pediatric dentist will face are the oral lesions. As much as I try to read or attend seminars, whenever you see a lesion, it’s never the same. I think if anyone wants to improve themselves, it should be in oral medicine.*”

[Participant 1 H S]

3. Diagnostic Uncertainty

Some participants encountered difficulties in diagnosing certain lesions due to their unknown nature or cause, making it challenging to establish a definitive diagnosis. In such cases, they listed differential diagnoses until confirmation could be obtained through further investigations, such as consultation with a surgeon or a biopsy, as noted by several respondents.

“*There was a lesion in the mandible, and it was multilocular. There were many differential diagnoses for the case, and it was difficult to diagnose, so it needed a biopsy.*”

[Participant 12 A S]

4. Rarity of Oral Lesions

Most participants had difficulty diagnosing or recognizing lesions because they rarely encounter such cases. This lack of exposure causes confusion when seeing a lesion for the first time and increases the risk of misdiagnosis; in some cases, there are no available references in textbooks.

“*These things are rare or we don’t see them daily like caries. So still, we might misdiagnose or miss some information that leads us to a good or proper diagnosis.*”

[Participant 6 R C]

C. Other Themes

5. Parental Attitude and Behavioral Challenges

Several pediatric dentists noted that parents’ unwillingness to answer questions, reluctance to cooperate with referrals for further investigations, or refusal of necessary treatments can pose significant challenges. Some quotes illustrating this theme include:

“*One challenge is convincing the family to go to the pediatric when they inform about GI problem to go to the pediatric physician and examine to see what’s happening for further examination and management. Most of the time they are not convinced to go and see a pediatric or maybe they are convinced but it’s hard or it’s expensive for them to go, especially if it’s not in governmental sector.*”

[Participant 4 H S]

6. Patient Age and Behavioral Challenges

Several participants reported that conducting examinations and managing even simple dental procedures are challenging in uncooperative children, especially in younger ones. They highlighted barriers such as difficulties in communication and a lack of patient cooperation, which often complicate routine care. Representative participant quotes are listed below:

“*As we know, the younger they are, the worse the cooperation can be. If he had dental trauma or phobia, it makes the case worse. The behavior of the patient will make the procedure difficult for me. Maybe the dental treatment that would be easy to do without sedation with a cooperative child, even if it was a biopsy, would be very challenging and difficult for a patient who is young age and severely uncooperative, whether in time or in the procedure itself.*”

[Participant 11 J S]

7. System-Level Barriers

Many participants believed that system-level barriers, particularly those related to the referral system, resource constraints, and teamwork and interdisciplinary communication challenges, heavily impacted the diagnosis and management of pediatric OFCs.

- Referral System Issues

Most pediatric dentists mentioned issues with the referral protocol to other departments and expressed concern about the time it takes to refer a patient through the system.

“*Having no slots to see the patient immediately, especially if it’s urgent or the doctor is not on the floor, no consultant, only the resident or the fellows are dealing with the cases. Sometimes the referral is missed. The patient got an appointment for referral, and he did not show up. We didn’t know that he didn’t go. From their side, they don’t know that the patient is urgent or he need to be seen, so they don’t book him again.*”

[Participant 8 RB C]

- Resource Constraints

Many participants highlighted that some hospitals lacked oral medicine specialists. They also noted limitations in the availability of certain diagnostic tools and materials. Representative participant quotes include:

“*Some hospitals and clinics don’t have an oral medicine department, so it will be difficult to do examination and diagnosis.*”

[Participant 12 A S]

- Teamwork and Interdisciplinary Communication Challenges

Some participants shared their experiences with the lack of a coordinated team, the resulting need to transfer patients to other hospitals, the absence of a team in the governmental sector, and problems with a lack of direct communication between the dental and medical departments.

“*There is no direct communication. I talk about private; for example, I don’t have the extension. I am in the dental center; there is no pediatrician. So, I must talk to the other clinic to know the extension and the working hours of the doctor. So, when there is no such direct access, I refer them to the ER or to a pediatric hospital.*”

[Participant 7 S C]

### 3.2. Results of Quantitative Phase (II)

#### Participant Characteristics

A total of 180 pediatric dentists completed the questionnaire (Table 3). Female participants comprised 110 (61.1%) of the sample. Participants were relatively evenly distributed according to years of professional experience, with 60 (33.3%) reporting 2–5 years of experience and another 60 (33.3%) reporting more than 10 years of experience. Specialists represented 69 (38.3%) of the participants, while 64 (35.6%) held board certification. Most participants graduated from Saudi Arabia (114; 63.3%).

Most participants encountered OFCs sometimes (every 2–4 months) (40.0%) and rated their training as 4 (36.7%). Most reported receiving both theoretical and clinical exposure (62.8%) and had encountered OFCs during the previous 12 months (77.2%). Regarding diagnosed cases, 67.2% reported diagnosing 0–3 cases, while 32.8% diagnosed 4–6 or more cases (Table 4).

The distribution of correct answers for the diagnosis and management of the cases is shown in Table 5. The highest diagnostic scores were observed for traumatic ulcers and hand, foot, and mouth disease, with 178 participants (98.9%) correctly identifying each condition. The correct management of a traumatic ulcer was identified by 176 participants (97.8%), whereas only 137 participants (76.1%) correctly selected the appropriate management for hand, foot, and mouth disease. Overall diagnostic and management scores were calculated for each participant based on the number of correct responses across the 10 cases and are presented as the mean ± SD and median, with scores ranging from 0 to 10.

Table 6 presents an analysis of the response frequency of the most helpful strategies in a question that allowed participants to select more than one answer. The most frequently reported strategy was gaining more clinical exposure and hands-on experience, with 118 responses (32.7%).

### 3.3. Triangulation

Triangulation was conducted to integrate and interpret the findings of both phases (Table 7). Triangulation offers several advantages by incorporating different methods, perspectives, or sources to support the interpretation of findings. It strengthens the study design by integrating quantitative and qualitative findings instead of depending on a single approach, allowing for a broader understanding of the research phenomenon and supporting a more comprehensive interpretation of the results [18].

## 4. Discussion

The present exploratory sequential mixed-methods study aimed to assess the diagnostic performance of pediatric dentists in identifying OFCs, explore the common barriers and challenges encountered during diagnosis, and develop an assessment tool to evaluate case-based diagnostic performance and associated diagnostic challenges. The rationale for starting with the qualitative phase was to explore pediatric dentists’ knowledge, experiences, barriers and approaches to identifying and diagnosing OFCs, particularly given the lack of previous studies assessing diagnostic performance in this population. Including certified pediatric dentists (specialists and consultants) from different centers and with varying years of experience across regions of Saudi Arabia provided a deeper understanding of the knowledge gaps, diagnostic approaches, and potential challenges and barriers encountered when diagnosing OFCs in children [19,20,21,22]. The findings from the qualitative phase were then used to develop an assessment tool that was validated for the subsequent quantitative phase. Combining quantitative and qualitative approaches in one study resulted in stronger and more comprehensive findings than using either method independently [23].

Our qualitative findings suggested that no clear difference in diagnostic performance was observed between specialists and consultants and that consultants with many years of experience may still make incorrect diagnoses. These findings are consistent with previous studies that show similar performance between specialists and general dentists or less-experienced clinicians in diagnosing OFCs [24]. On the other hand, other studies have reported that specialists showed better diagnostic performance scores than general dental practitioners in the diagnosis of OFCs [25,26]. This may indicate factors beyond clinical rank and years of experience, such as clinical exposure to a broad spectrum of lesions and variability in continuing education and training opportunities [27].

In the present study, although participants reported using an institutional electronic referral system, many still relied on phone-based referrals to reduce appointment delays. This result is supported by previous reports, where referral communication for oral potentially malignant lesions or malignancy included written electronic correspondence and patient-carried information, whereas referral appointments were also organized through telephone communication [28]. In contrast, previous studies have shown that teleconsultation significantly reduced unnecessary in-person referrals by 35% to 80% and shortened waiting times through structured digital platforms that facilitate the exchange of clinical data and images [29].

Dentist-related factors, particularly insufficient training and experience, emerged as an important finding in this study. This challenge has been reported by several studies as contributing to the difficulty of diagnosing OFCs due to a lack of skills or confidence, often attributed to insufficient training and experience and lack of adequate knowledge [24,30,31,32]. This may result in delayed referrals or treatment.

In addition to dentist-related factors, lesion-related challenges were also identified in this study, including the complexity and atypical presentation of orofacial lesions, diagnostic uncertainty, and the rarity of oral lesions. These findings are consistent with previous studies showing that nonspecific features and variability in clinical presentations make diagnosis challenging and increase the risk of misdiagnosis and improper treatment [33,34,35]. Similarly, it has been reported that oral ulceration may have multiple etiologies, with variations in nature and clinical presentation making accurate diagnosis particularly challenging [9,36]. Furthermore, it has been reported that the low incidence of oral lesions may further contribute to reduced familiarity among practitioners, which can negatively affect both timely diagnosis and appropriate management strategies [37].

Although participants reported several challenges and barriers to diagnosis, the present study did not assess whether these factors were associated with lower diagnostic performance. Therefore, these findings should be interpreted as participants’ perceived barriers to diagnosis rather than as confirmed determinants of diagnostic performance.

Participants in the present study emphasized clinical exposure and hands-on experience as essential for improving diagnostic and management skills. This is consistent with evidence that supervised clinical experience helps translate theoretical knowledge into practice and enhances diagnostic competence and confidence, while continuing professional development (CPD) alone may be insufficient without adequate clinical exposure [38,39]. Hussein et al. similarly highlighted the need for structured curricula integrating theoretical and practical training to address educational gaps in diagnosing and managing OFCs [40]. Strengthening oral medicine and pathology education, incorporating relevant medical content, and providing CPD can further improve dentists’ diagnostic performance [24,41].

Emerging artificial intelligence (AI) technologies may offer complementary approaches to some of the challenges identified in the present study. Large language models (LLMs) have demonstrated potential in reducing the workload associated with preparing educational materials [42]. An LLM-based Anki tool substantially reduced flashcard preparation time and cognitive load among dental students, potentially facilitating self-directed learning and allowing greater focus on conceptual understanding [42]. Furthermore, vision–language models (VLMs) have shown potential as zero-shot tools for supporting the interpretation of condylar osseous changes on CBCT and generating structured diagnostic reports [43]. Such tools may provide supplementary support for learners with limited radiological training, although expert supervision and further clinical validation remain essential [43]. These developments highlight opportunities for integrating AI-assisted learning and diagnostic support into dental education to complement conventional training and address identified educational barriers.

The findings of this study have important clinical implications. Enhancing practical clinical training, promoting CPD, and establishing clear diagnostic protocols may help reduce variability in diagnostic performance. Furthermore, strengthening interdisciplinary collaboration among pediatric dentists, oral medicine specialists, and oral pathologists may facilitate more accurate diagnoses and ultimately improve patient outcomes.

Several strengths of this study should be acknowledged. The mixed-method design provided both breadth and depth by integrating questionnaire data with interview findings, enhancing understanding of and confidence in the conclusions [44,45]. Triangulation between the two phases further strengthened the validity and credibility of the findings [46]. Credibility was ensured through multiple interviews, member checking, established methods, an appropriate study design, and interviewer training. Transferability was supported by purposeful sampling of specialists and consultants from multiple institutions and regions across Saudi Arabia, as well as inclusion of participants who had encountered OFCs within the previous year to minimize recall bias. Dependability was maintained through consistent research procedures and thorough documentation throughout the study. Confirmability was strengthened through piloting and revising the interview guide, maintaining written field notes, and comparing findings with existing literature. Additionally, the questionnaire’s wide geographical coverage and large sample of pediatric dentists across Saudi Arabia enhanced the diversity and representativeness of the study population.

This study has some limitations. The qualitative phase often involved lengthy interviews lasting more than 30 min, which could lead to participant fatigue. Moreover, consultants were often busy and answered phone calls during the interviews. Furthermore, some online interviews experienced technical challenges, leading to interruptions. The interviewer was a single female resident in pediatric dentistry with a background in OFCs, which may have introduced reflexivity bias by influencing participants’ responses throughout the qualitative phase [47,48,49]. Additionally, the quantitative phase evaluated diagnostic performance using hypothetical case scenarios rather than real-time clinical encounters, which may not accurately reflect true clinical performance under pressure. The exclusion of difficult, rare and syndromic conditions may reduce the applicability of the findings to the specialist rank. Furthermore, the online administration of the questionnaire without supervision may have allowed participants to seek assistance from external sources, such as ChatGPT or Google, when completing the questionnaire, potentially inflating the diagnostic performance scores. Finally, the study findings and validated assessment tool are derived exclusively from participants across Saudi Arabia, limiting their applicability to international dental practice settings.

Future research should focus on having participants complete the questionnaire on the spot to ensure that no external sources are used and to ensure the accuracy of the results. Additionally, investigating the impact of digital diagnostic tools and AI on the identification of OFCs may provide valuable insights into enhancing clinical practice.

## 5. Conclusions

This mixed-methods study resulted in the development of a tool for assessing pediatric dentists’ diagnostic performance and associated diagnostic challenges. The qualitative findings identified key barriers, including insufficient training or experience, lesion complexity and atypical presentation, diagnostic uncertainty, behavioral challenges, system-level barriers, and lesion rarity. The quantitative findings provided evidence supporting the questionnaire’s validity for its intended purpose. The questionnaire findings also indicated high diagnostic performance and management scores among pediatric dentists in Saudi Arabia in identifying pediatric OFCs. The tool is specific to pediatric OFCs in Saudi Arabia and may not be generalizable to other settings. The findings highlight the importance of improving hands-on training and interdisciplinary collaboration, including joint clinics with oral medicine physicians and oral and maxillofacial surgeons to support accurate diagnosis and effective management of pediatric OFCs.

## Figures and Tables

**Table 1:** Demographic characteristics of the interviewed participants.

Variable	Category	Frequency (n)	Percentage (%)
Participant Gender	Male	2	15.4%
	Female	11	84.6%
Participant Rank	Specialist	6	46.2%
	Consultant	7	53.8%
Participant Graduation Country	Saudi Arabia	10	76.9%
	United States	3	23.1%
Participant Level of Education	Master’s Degree	2	15.4%
	Board Certification	9	69.2%
	PhD	2	15.4%
Workplace	Jeddah	9	69.2%
	Outside Jeddah	4	30.8%
Participant Years of Experience	Less than 5 Years	2	15.4%
	5–10 Years	7	53.8%
	More than 10 Years	4	30.8%

**Table 2:** Diagnostic performance by pediatric dentists’ rank.

Diagnostic Performance	Specialists n (%)	Consultants n (%)	Total n (%)
Correct diagnosis	4 (30.8%)	4 (30.8%)	8 (61.5%)
Wrong diagnosis	2 (15.4%)	3 (23.1%)	5 (38.5%)
Total	6 (46.2%)	7 (53.8%)	13 (100%)

**Table 3:** Sociodemographic and professional characteristics of participants (N = 180).

Variable	Category	Frequency (n)	Percentage (%)
Gender	Male	70	38.9
	Female	110	61.1
Years of experience	≤1 year	12	6.7
	2–5 years	60	33.3
	6–10 years	48	26.7
	>10 years	60	33.3
Clinical ranking	Resident	54	30.0
	Specialist	69	38.3
	Consultant	57	31.7
Educational level	Residency	46	25.6
	Master’s degree	42	23.3
	Board certification	64	35.6
	PhD	28	15.6
Graduation country	Saudi Arabia	114	63.3
	North America	17	9.4
	Europe	17	9.4
	Other	32	17.8
Practice setting	Private	53	29.4
	Academic hospital/institute	52	28.9
	Government hospital	75	41.7
Level of practice	Primary	18	10.0
	Secondary	86	47.8
	Tertiary	76	42.2
Location of practice	Western	93	51.7
	Eastern	19	10.6
	Central	47	26.1
	Northern	10	5.6
	Southern	11	6.1
Practice nature	Urban	172	95.6
	Rural	8	4.4
Working time	Full-time	167	92.8
	Part-time	13	7.2
Number of patients seen per week	≤12	52	28.9
	13–25	51	28.3
	26–38	38	21.1
	39–50	26	14.4
	>50	13	7.2

**Table 4:** Participants’ clinical exposure to OFCs (N = 180).

Variable	Category	Frequency (n)	Percentage (%)
Frequency of encountering OFCs	Always	7	3.9
	Often (monthly)	49	27.2
	Sometimes (every 2–4 months)	72	40.0
	Rare (once a year)	45	25.0
	Never	7	3.9
Training received	Very little	13	7.2
	2	17	9.4
	Neutral	63	35.0
	4	66	36.7
	Extensive	21	11.7
Nature of exposure	Theoretical	56	31.1
	Clinical	11	6.1
	Both	113	62.8
Exposure during the previous 12 months	Yes	139	77.2
	No	41	22.8
Number of diagnosed cases	0–3	121	67.2%
	4–6 or more	59	32.8%

**Table 5:** Diagnostic and management performance across clinical cases (N = 180).

Case Number	Diagnosis	Management	Answers of Correct Diagnosis n (%)	Answers of Correct Management n (%)
1.	Recurrent aphthous-like ulcer	Reassurance and referral to pediatrician for laboratory investigations	164 (91.1%)	175 (97.2%)
2.	Acute herpetic gingivostomatitis	Palliative, supportive, and symptomatic care	136 (75.6%)	151 (83.9%)
3.	Mucocele	Surgical excision and/or reassurance	170 (94.4%)	175 (97.2%)
4.	Traumatic ulcers	Reassurance and palliative care	178 (98.9%)	176 (97.8%)
5.	Eruption cyst	Reassure parent and follow-up	172 (95.6%)	170 (94.4%)
6.	Geographic tongue	Reassurance	168 (93.3%)	160 (88.9%)
7.	Oral candidiasis (oral thrush)	Topical antifungal	163 (90.6%)	154 (85.6%)
8.	Pyogenic granuloma	Laser excision	138 (76.7%)	163 (90.6%)
9.	Irritational fibroma	Laser excision	157 (87.2%)	127 (70.6%)
10.	Hand, foot, and mouth disease	Palliative, supportive, and symptomatic care	178 (98.9%)	137 (76.1%)
	Overall participant-level score (out of 10)		Mean ± SD = 9.03 ± 1.16; median = 9	Mean ± SD = 8.84 ± 1.32; median = 9

**Table 6:** Participants’ responses on helpful strategies (multiple responses allowed) (N = 361).

Helpful Strategy	Frequency (N)	Percentage (%)
Gain more clinical exposure and hands-on experience	118	32.7%
CPD courses on pediatric orofacial conditions	76	21.1%
Self-education by reading and reviewing	81	22.4%
Collaborate with specialists and use referral systems	86	23.8%
Total selections	361	100.0%

N = Total number of responses; multiple responses were allowed.

**Table 7:** Triangulation of qualitative and quantitative phases.

	Qualitative Findings	Quantitative Findings	Triangulation
I	Participants described documenting orofacial cases and engaging in clinical case discussions. “*As a clinical instructor, we sometimes face these cases in the clinic. So, we document, we take history, we go back and read. Sometimes we have discussions in our center to review the most common based on what we see.*” [Participants 4 H S]	The most helpful strategies for improving diagnostic performance were gaining more clinical exposure and hands-on experience	Convergence: Both findings highlighted clinical exposure as an important aspect of diagnostic competence.
II	Participants reported having adequate knowledge; however, limited clinical exposure impacted their clinical skills in diagnosing orofacial cases. “*All of that we studied didactically in books; we reviewed cases in photos, but definitely the challenge is that we don’t see it in the clinic, so it doesn’t get reinforced.*” [Participant 9 I C]	The area most identified as lacking was limited clinical exposure to pediatric orofacial lesions.	Convergence: Both findings identified limited clinical exposure as a perceived limitation in diagnosing pediatric OFCs.
III	Participants reported rarely encountering pediatric orofacial lesions in practice. “*Very rare honestly, how many do we see? it’s not daily or weekly. It’s monthly. You know, every month you can say we can see one case, for example. Sometimes more than one month*”. [Participants 5 O C]	The area most identified as lacking was limited clinical exposure to pediatric orofacial lesions.	Complementarity: The qualitative findings explained that limited clinical exposure to pediatric orofacial lesions was due to the rarity of encountering such cases in clinical practice.
IV	Participants described atypical lesion presentations and uncommon cases as contributing to diagnostic difficulty and the need to seek specialist consultation. “*One of the cases that came to me had very unclear features of a mucocele. It was a bit larger and didn’t appear as fluid as typical mucoceles. I had to consult my friend to reach a diagnosis. It turned out to be a certain type of mucocele that is not commonly seen.*” [Participant 10 A S]	The most observed challenge was the complexity and atypical presentation of oral lesions.	Convergence: Both findings identified complex and atypical lesion presentations as challenges in diagnosis.
V	Participants described the referral system, multidisciplinary communication, and consultation. “*I do it through the system and follow the university’s protocol. It is very clear. When I make a referral, as a consultant, I do not rely solely on the residents or the system, so that the patient does not get lost in the process. I also communicate the referral to the maxillofacial consultant by phone.*” [Participant 2 N C]	Referring the case to specialists within or outside the institution.	Complementarity: The qualitative findings provided examples of referral and multidisciplinary consultation, while the quantitative findings indicated that referring cases to specialists was a commonly selected approach.

## Data Availability

The original contributions presented in this study are included in the article. Further inquiries can be directed to the corresponding author.

## Abbreviations

The following abbreviations are used in this manuscript:

OFCs	Orofacial conditions
CPD	Continuing professional development
CBCT	Cone-beam computed tomography
AI	Artificial intelligence
LLMs	Large language models
VLMs	vision–language models

## Appendix A

**Table A1:** Content of the interview guide.

**Section 1: Introduction and Consent**
**Section 2: Demographic Information**
1. What is your rank? 2. What is your clinical experience? How many years? 3. Which country did you graduate from, and what are your degrees? 4. What is your occupation? Prompts: “the place of the work, working hours” 5. What is your current workplace? Prompts: (university hospital, private clinic, public hospital, etc.) 6. Please describe the extent and nature of your training or clinical exposure to oral pathology during your residency program.
**Section 3: Clinical Experience with Orofacial Conditions**
7. How many clinics do you have weekly? 8. How many patients do you see each month? 9. How frequently do you encounter orofacial conditions? 10. What are the three most common lesions you have encountered in the last year? (child’s complaint, diagnosis, confidence in diagnosis, differential diagnosis, referral decision, diagnostic methods, management). 11. Describe a case where the diagnosis was challenging. 12. What is your referral system?
**Section 4: Diagnostic and Management Process (Case-Based Scenario)**
13. (Provisional diagnosis, differential diagnoses, management plan).
**Section 5: Challenges and Barriers**
14. What are the main challenges you faced, or do you think that pediatric dentists may face when diagnosing or managing orofacial conditions in children? Prompt: (lack of training, overlapping clinical features, limited access to diagnostic tools, time or resource constraints, children’s behavior and age of the child).
**Section 6: Support and Improvement**
15. In your opinion, what knowledge or skills are most lacking among pediatric dentists in this area? 16. How do you usually update your knowledge regarding pediatric orofacial conditions? 17. What kind of tools or resources would help you improve your diagnostic performance (e.g., clinical guidelines, decision trees, diagnostic apps, photo-based atlases)? 18. Do you think having a structured checklist or diagnostic tool would be useful in your routine practice? For example, a list to guide you in identifying the type of lesion and deciding which investigations to request. Why or why not?” 19. What recommendations would you give to improve pediatric dentists’ ability to diagnose and manage these conditions more effectively?

## Appendix B

**Table A2:** An online questionnaire.

Do You Agree to Participate in This Questionnaire?		☐ Yes	☐ No
Section 1: The Following Questions Relate to Your Personal and Professional Background			
1.	Gender	☐ Male ☐ Female	
2.	Years of experience	☐ 1 year or less ☐ 2–5 years ☐ 6–10 years ☐ More than 10 years	
3.	What is your clinical ranking in pediatric dentistry?	☐ Resident ☐ Specialist ☐ Consultant	
4.	Highest education level?	☐ Residency ☐ Master’s degree ☐ Board certified/clinical certificate in pediatric dentistry ☐ Doctorate/PhD	
5.	Graduation country of your highest degree	☐ Saudi Arabia ☐ North America ☐ Europe ☐ Other	
6.	Practice (please choose the primary one)	☐ Private practice ☐ Academic hospital/institute ☐ Government hospital	
7.	Level of practice	☐ Primary health care (basic and preventive care) ☐ Secondary health care (specialist care) ☐ Tertiary health care (highly specialized and advanced care)	
8.	Location of practice	☐ Western region ☐ Eastern region ☐ Central region ☐ Northern region ☐ Southern region	
9.	Practice nature	☐ Urban (city) ☐ Rural (villages)	
10.	Your working hours (please choose the primary one)	☐ Full-time ☐ Part-time	
11.	On average, how many patients do you see per week?	☐ 12 or less ☐ 13–25 ☐ 26–38 ☐ 39–50 ☐ More than 50	
12.	On average, how frequently do you encounter orofacial conditions/disorders?	☐ Always (weekly) ☐ Often (monthly) ☐ Sometimes (every 2–4 months) ☐ Rarely (once a year) ☐ Never	
13.	To what extent do you feel your dental education trained you to diagnose orofacial conditions? 1: Very little training 5: Extensive training	☐ 1 ☐ 2 ☐ 3 ☐ 4 ☐ 5	
14.	What was the nature of your exposure and training in oral medicine during residency?	☐ Theoretical ☐ Clinical exposure ☐ Both	
15.	Have you been exposed to orofacial conditions in the last 12 months?	☐ Yes ☐ No	
16.	How many cases of orofacial conditions have you diagnosed correctly in the last 12 months?	☐ 0–3 ☐ 4–6 or more	
Section 2: There Will Be a Series of Clinical Pictures. Please Answer the Following Questions			
17.	Case (1): A 6-year-old girl presented with recurrent painful oral ulcers associated with discomfort during eating. She had a history of recurrent abdominal pain suggestive of an underlying gastrointestinal disorder. What is your most probable diagnosis?	☐ Traumatic ulcer ☐ Recurrent intraoral herpes ☐ Recurrent aphthous-like ulcer ☐ Primary herpetic gingivostomatitis	
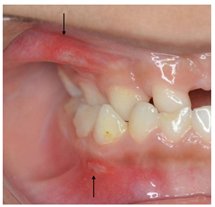
18.	What is the primary action you would take to manage the above case?	☐ Reassurance, home care instructions, and referral to a pediatrician for laboratory investigations ☐ Topical antiviral ☐ Systemic antibiotic ☐ Biopsy	
19.	Case (2): A 5-year-old otherwise healthy female patient presented with painful oral ulcers that began yesterday. She has difficulty opening her mouth and is experiencing challenges with eating. She also has a history of fever and lymphadenopathy. No lesions were noted elsewhere. What is your most probable diagnosis?	☐ Herpangina ☐ Recurrent aphthous ulcer ☐ Hand, foot and mouth disease ☐ Acute herpetic gingivostomatitis	
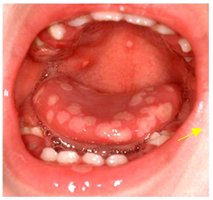
20.	What is your management of the above-mentioned case?	☐ Palliative, supportive, and symptomatic care ☐ Topical corticosteroids ☐ Topical antiviral ☐ Referral to a pediatrician for laboratory investigations	
21.	Case (3): A 5-year-old otherwise healthy male patient presented with a diameter of 2 mm, painless, fluctuant, fluid-filled swelling on the lower lip, noticed by the parent after playing football. What is your most probable diagnosis?	☐ Ranula ☐ Fibroma ☐ Mucocele ☐ Molluscum contagiosum	
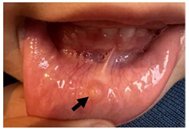
22.	What is your management of the above-mentioned case? (You may choose more than one.)	☐ Reassurance and follow-up ☐ Antibiotic therapy ☐ Intralesional corticosteroids ☐ Surgical excision	
23.	Case (4): A 7-year-old otherwise healthy male patient presented with a painful oral ulcer following recent dental work. What is your most probable diagnosis?	☐ Recurrent herpes labialis ☐ Traumatic ulcer ☐ Contact stomatitis ☐ Recurrent aphthous stomatitis	
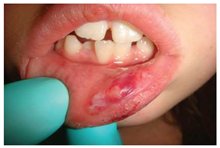
24.	What is your management of the above-mentioned case?	☐ Reassurance and palliative care ☐ Soft tissue radiograph ☐ Topical antiviral ☐ Biopsy	
25.	Case (5): An 8-month-old female infant was brought by her mother with a concern about a swelling in the upper anterior alveolar ridge. The mother reported that the swelling had been present for two days and stated that the child appeared to be in pain. What is your most probable diagnosis?	☐ Epstein pearls ☐ Eruption cyst ☐ Hemangioma ☐ Congenital epulis	
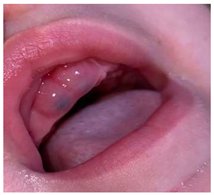
26.	What is your management of the above-mentioned case?	☐ Parental reassurance and follow-up ☐ Referral to a pediatrician for laboratory investigations ☐ Laser ablation ☐ Surgical exposure	
27.	Case (6): An 8-year-old, otherwise healthy male patient presented for a regular check-up. Asymptomatic, irregular erythematous areas on the dorsal surface of the tongue, surrounded by a white border, were incidentally noted during the clinical examination. What is your most probable diagnosis?	☐ Lichenoid reaction ☐ Oral candidiasis ☐ Oral lichen planus ☐ Geographic tongue	
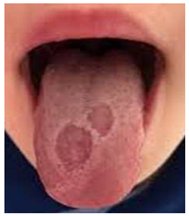
28.	What is your management of the above-mentioned case?	☐ Reassurance ☐ Topical corticosteroids ☐ Topical antifungal ☐ Laser ablation	
29.	Case (7): A 3-week-old exclusively breastfed infant presented with a 5-day history of white patches on the tongue and buccal mucosa that left an erythematous base after being wiped off. What is your most probable diagnosis?	☐ Leukoplakia ☐ Oral lichen planus ☐ Milk residue on oral mucosa ☐ Oral candidiasis (oral thrush)	
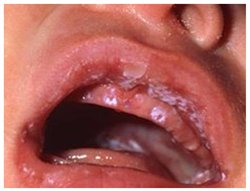
30.	What is your management of the above-mentioned case?	☐ Reassurance ☐ Topical corticosteroids ☐ Topical antifungal ☐ Laser ablation	
31.	Case (8): An 11-year-old otherwise healthy male patient presented for a dental check-up. A reddish, raised, pedunculated gingival lesion was noted, which was ulcerated, soft to firm and bled easily when brushed. What is your most probable diagnosis?	☐ Fibroma ☐ Pyogenic granuloma ☐ Gingival hyperplasia ☐ Peripheral giant cell granuloma	
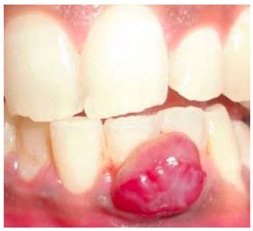
32.	What is your management of the above-mentioned case?	☐ Reassurance ☐ Topical corticosteroids ☐ Intralesional corticosteroids ☐ Laser excision	
33.	Case (9): A 7-year-old girl with a history of tongue biting presented with a small, painless, firm, pink nodule on her tongue. What is your most probable diagnosis?	☐ Mucocele ☐ Irritational fibroma ☐ Pyogenic granuloma ☐ Squamous papilloma	
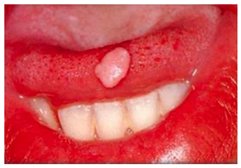
34.	What is your management of the above-mentioned case?	☐ Reassurance ☐ Topical corticosteroids ☐ Topical antiviral ☐ Laser excision	
35.	Case (10): A 3-year-old male child presented with fever, irritability, and pain. Clinical examination revealed erythematous papules and vesicles on the palms, along with multiple oral ulcers. What is your most probable diagnosis?	☐ Varicella (chickenpox) ☐ Allergic contact dermatitis ☐ Hand, foot, and mouth disease ☐ Primary herpetic gingivostomatitis	
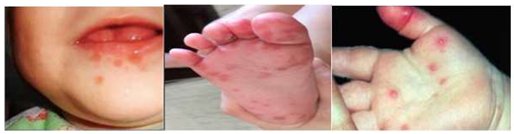
36.	What is your management of the above-mentioned case?	☐ Palliative, supportive, and symptomatic care ☐ Topical antiviral ☐ Topical corticosteroids ☐ Referral to a pediatrician for laboratory investigations	
Section 3: The Following Questions Relate to Your Opinions and Experience.			
37.	What are the most common challenges you have encountered when diagnosing or managing orofacial conditions in children?	☐ Insufficient training or experience ☐ Complexity and atypical presentation of orofacial lesions ☐ System-level barriers (e.g., referral processes, resource constraints, teamwork and interdisciplinary communication) ☐ Child age and behavior challenges ☐ Parental attitude and behavior challenges ☐ Diagnostic uncertainty ☐ Rarity of oral lesions ☐ Other	
38.	Does your center/clinic have an oral medicine specialist or consultant?	☐ Yes ☐ No	
39.	When you encounter an orofacial condition, what is your preferred course of action?	☐ Follow-up ☐ Manage the case by yourself ☐ Refer the case to a specialist within your institute ☐ Refer the case to a specialist outside your institute ☐ Other	
40.	Which of the following strategies do you consider most helpful for improving your ability in the diagnosis and management of orofacial conditions?	☐ Gain more clinical exposure and hands-on experience with a variety of cases ☐ CPD courses on pediatric oral conditions ☐ Self-education by reading and reviewing textbooks, case reports, and scientific literature ☐ Collaborate with specialists and use referral systems ☐ Other	
41.	What knowledge or skills are most lacking among pediatric dentists in this area?	☐ Limited clinical exposure to pediatric orofacial lesions ☐ Weak systematic diagnostic approach ☐ Insufficient training in oral medicine during your specialty training ☐ Inadequate ongoing education and refresher training in this area ☐ Other	
